# A study of students’ learning perceptions and behaviors in remote STEM programming education

**DOI:** 10.3389/fpsyg.2022.962984

**Published:** 2022-10-06

**Authors:** Yu-Sheng Su, Ching-Yao Chang, Cheng-Hsin Wang, Chin-Feng Lai

**Affiliations:** ^1^Department of Computer Science and Engineering, National Taiwan Ocean University, Keelung City, Taiwan; ^2^Department of Computer Science and Engineering, National Central University, Taoyuan City, Taiwan; ^3^Department of Engineering Science, National Cheng Kung University, Tainan City, Taiwan

**Keywords:** remote STEM programming, STEM education, learning performance, learning perceptions, learning behaviors

## Abstract

In recent years, STEM education has developed students’ fundamental subject knowledge, and has allowed students to integrate STEM cross-domain knowledge. Universities emphasize innovative thinking, practice and application, problem-solving, and teamwork to nurture students while learning STEM cross-domain knowledge development in remote STEM program education. When students take online STEM programs, they may encounter unanswered questions and may give up on trying to solve them. Therefore, this study proposed a problem-based learning approach with an online programming system integrated into an online STEM programming course. To help students solve the past programming assignments, the assignments were simplified, decomposed, and reorganized. The teacher guided the students to understand the STEM programming problems and taught them to use appropriate problem-solving skills to motivate them to complete the STEM programming assignments. The experiment was conducted with students in an online STEM programming course at a university in northern Taiwan. In the experimental activities, we used a problem-based learning approach for the online STEM programming activity. The problem-based learning method can be divided into four steps, namely stating the problem, understanding the problem, developing a solution plan, and executing the plan, reflecting, and debugging. This study used a problem-based learning approach and an online programming system integrated into a STEM programming curriculum to explore the differences in students’ perceptions of STEM learning, learning outcomes, and learning behaviors. The experimental results found a significant difference between students’ prior knowledge and learning outcomes. Students showed significant gains in learning the STEM programming content using the problem-based learning approach and the online programming system. In the analysis of their STEM learning perceptions, we found that there were significant differences in students’ responses for each dimension. This shows that using the problem-based learning approach with the online programming system helped students learn the course content. The analysis of students’ behaviors in answering the STEM programming assignments indicated that some students had the habit of taking notes. This helped them to easily associate and integrate STEM cross-domain knowledge with what they had learned in the online course, and enhanced their ability to implement STEM programs. In addition, students could take the initiative and focus on repeatedly watching the teacher solve the material in the online course. Students could try different solving plans to pass the code validation of the STEM programming assignments. This revealed that students wanted to complete the STEM programming assignments to achieve good learning performance.

## Introduction

In 1986, the United States National Research Council proposed the STEM education agenda of integrating Science, Technology, Engineering, and Mathematics. The four core elements of STEM education are all interrelated. The goal is to train students to become cross-disciplinary experts, like mathematicians, scientists, engineers, or technologists. STEM education is helpful for students to think about cross-disciplinary knowledge and to learn cross-disciplinary professional skills (Lou et al., 2011; Ritz and Fan, 2015; Newhouse, 2017; Lai et al., 2021). For example, the equations of mathematics are often used in science, technology, and engineering, and mathematics is used to solve problems in science, technology, and engineering. Ritz and Fan (2015) found that most people believe that it is important to explore the meaning of STEM education and to propose corresponding cross-disciplinary education directions. This will help learners to develop appropriate cross-disciplinary talents. Therefore, every country in the world attaches great importance to STEM education.

In 2014, the National Science Board of America emphasized that STEM education develops students’ ability to integrate cross-disciplinary knowledge and learn hands-on problem-solving skills (A Lou et al., 2011; Newhouse, 2017). Achilleos et al. (2019) organized a STEM programming competition in America, which was conducted through a social platform with online STEM education themes. This online STEM activity enhanced students’ motivation to take STEM programs. At the same time, students can understand the positive benefits of STEM education through their own learning of STEM programs. In the White Paper on Creativity Education, the Ministry of Education (2003) mentioned expanding the vision of education and developing the characteristics of each school by focusing on an innovative operating learning environment and lively teaching atmosphere. Universities are gradually developing a series of STEM courses, with the aim of learning from the European and American strategies of independent growth learning and adapting to the talents and abilities of the students (Wing, 2006; Shute et al., 2017; Ministry of Education, 2019). Universities emphasize students’ high-level thinking, practice and application, problem-solving, and teamwork to foster the integration of STEM cross-domain knowledge into STEM program development.

Previous studies (Su et al., 2017; Calvo et al., 2018; Achilleos et al., 2019) have attempted to integrate STEM education concepts into online program development and applications. These studies found that STEM education helps to develop students’ cross-disciplinary online programming skills. Achilleos et al. (2019) argued that students intuitively perform cross-domain programming rather than applying STEM cross-domain knowledge to complete their programs during the implementation of cross-domain online programming assignments. This finding revealed that students lacked the concept of STEM cross-domain knowledge, resulting in poor results in implementing cross-curricular online programs. Calvo et al. (2018) suggested that teachers use the NXC (C-like programming language for the NXT brick) programming tool to teach students to assemble the LEGO Mindstorms kit, thus allowing students to learn the concepts of STEM cross-curricular knowledge. The results of their study found that the course pass rate was high, dropouts were reduced, and students did participate in the online course and showed positive attitudes.

When students solve online programming assignments, they may encounter unanswered questions which may lead them to give up on solving the assignments. To address this problem, Tang et al. (2020) used a problem-based learning teaching strategy in which teachers taught students to learn how to solve programming assignments, which in turn enhanced students’ willingness to solve the assignments. Previous studies (Hung, 2008; Ismail et al., 2010; Lou et al., 2011; Schmidt et al., 2011; Bédard et al., 2012; Tseng et al., 2013; Uysal, 2014; Charlton and Avramides, 2016; Dos Santos, 2017; Mutiawani et al., 2017; Tang et al., 2020) have confirmed that the problem-based learning method has clear steps for learning. It also enhances students’ willingness to take the initiative in learning. The teacher provides the students with specific and clear objectives for the assignment, and the teacher appropriately assists and reminds the students during the problem-solving process. Bicer et al. (2015) used a problem-based learning approach to integrate teaching activities into science and mathematics classes. The results of their study showed that students in science and math classes effectively mastered the vocabulary, which is evidence that problem-based learning is a beneficial instructional approach. Chonkaew et al. (2016) explored the integration of a problem-based learning approach into a high school chemistry curriculum to explore students’ attitudes toward science learning and analytical thinking skills. They found that the problem-based learning approach to chemistry laboratory activities was successful in developing students’ analytical thinking and problem-solving skills. Gomoll et al. (2016) explored the use of a problem-based learning approach for students to integrate into a robotic programming curriculum, allowing students to take back ownership of their learning and students to be willingly engaged in their learning. The problem-based learning approach results in a more significant willingness to learn than the traditional teaching method. For example, when solving programming assignments, students know the objective of the assignment through the problem-based learning approach, and they dare to face programming errors and find possible solutions. LaForce et al. (2017) used a questionnaire to survey high school students about their learning perceptions. The results showed that students’ ratings of problem-based learning were related to interest in pursuing a STEM career, intrinsic motivation in science, and students’ beliefs about their abilities in science and mathematics. Lou et al. (2011) found that the problem-based learning approach influenced students’ attitudes toward learning STEM cross-domain knowledge. The experimental results indicated that the combination of teaching and learning approaches and STEM cross-domain knowledge enhanced learning outcomes, and that the resulting learning outcomes affected students’ attitudes toward future career pursuits. Su et al. (2014) proposed that a problem-based learning approach is integrated into an online programming course, and the implementation process of the problem-based learning method can be divided into four steps. Step 1 is stating the problem, step 2 is understanding the problem, step 3 is developing a solution plan, and step 4 is executing the plan, reflecting, and debugging. The results demonstrate that the integration of problem-based learning into a programming course can diversify the presentation of knowledge content and attempt to break through the current challenges that the online programming course may face. Therefore, this study used Su et al. (2014) proposed problem-based learning approach integrated into an online STEM programming curriculum to propose an appropriate teaching method. The problem-based learning approach interacts with STEM cross-domain knowledge and programming development. The mechanism incorporates STEM cross-domain knowledge into the online STEM programming curriculum, thereby enhancing students’ STEM knowledge and programming skills.

In this study, we proposed a problem-based learning approach and an online programming system integrated into the STEM programming activity. By integrating the problem-based learning approach with the online programming system, teachers can diversify the teaching content to enhance students’ collaboration and STEM cross-domain programming skills. In this experiment, the subjects were students from an online STEM programming course at a university in Taiwan. In the experimental activity, we used a four-step problem-based learning approach, namely stating the problem, understanding the problem, developing a solution plan, and executing the plan, reflecting, and debugging. This study explored the differences in students’ perceptions of STEM learning, learning outcomes, and learning behaviors when using the problem-based learning approach with the online programming system integrated into the online STEM programming curriculum. The study addressed the following research questions:

Q1: What are the differences between students’ prior knowledge and learning performance?

Q2: What are the differences between students’ learning perceptions?

Q3: What are the differences between students’ behaviors in answering STEM programming assignments?

## Methodology

### Participants

In this experiment, the subjects were 20 students from a university in northern Taiwan. This study was derived from a college elective course that was open to students in third grade and above to take the online STEM programming course. All students agreed and volunteered to participate in the experimental activity. Each student had a computer to use during class, and all students had basic experience of using computers.

### Learning materials

The learning materials were developed by STEM programming experts (Eckel and Allison, 2003). The learning objective of the learning materials was to provide students with technical knowledge of STEM programming and to develop their STEM cross-domain knowledge and programming skills.

The course was designed as a series of learning materials. The learning materials included basic concepts of STEM education, basic programming knowledge in C/C++, and STEM programming assignments. STEM cross-domain knowledge covers the basic concepts of science, technology, engineering, and mathematics. The teacher used STEM cross-domain knowledge combined with problem-based learning to design STEM programming assignments. Based on the scientific concept and programming inquiry, we use the programming knowledge of data types and strings to develop the scientific conceptual programming assignment for the number of consecutive salt-based repetitions of genes. Based on the design of programs with new technologies, we use the programming knowledge of iterators and generalized algorithmic functions to develop the technology conceptual programming assignment for old cell phones. Based on the finished structure and engineering thinking, we applied the programming knowledge of class inheritance and virtual functions to develop the engineering conceptual programming assignment for the circuit analysis. Based on computational mathematical thinking, we used the programmatic knowledge of operator overloading to develop the mathematical conceptual programming assignment for the Inverse matrix calculator.

### Procedure

**Figure 1:**
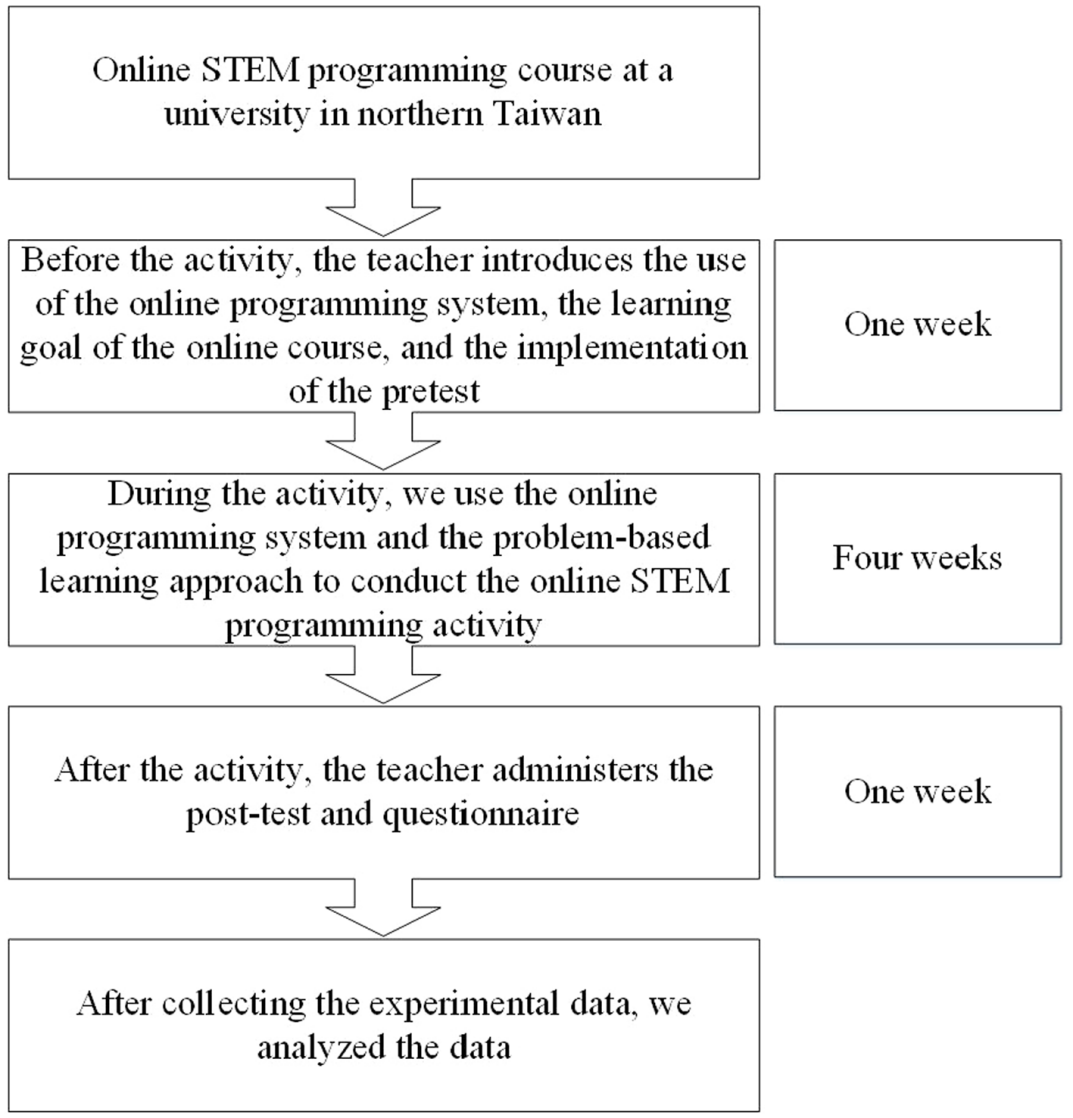
Experimental procedure.

During this experimental period, the COVID-19 outbreak occurred. The experiment was conducted for 6 weeks, once a week for 100 min, as shown in Figure 1.

In the first week, the teacher introduced the use of the online programming system and how to conduct the experimental activity. The teacher explained the learning objectives and assignments for the activity. Next, the teacher administered a pretest to understand the students’ prior knowledge level. The teacher designed the STEM programming materials, and applied the learning materials in class.

**Figure 2:**
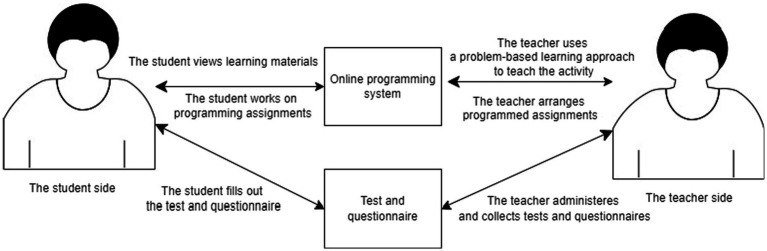
The online programming system and the problem-based learning method for the STEM programming administers.

During the experimental activity, we conducted a 4-week experimental STEM programming curriculum using an online programming system and a problem-based learning approach, with each weekly session lasting 100 min. The teacher taught the cross-domain knowledge of STEM programs in the classroom, and then assigned the STEM programming assignments to the students, who completed the assignments in the classroom. These assignments included science (S), technology (T), engineering (E), and mathematics (M) concepts, such as the number of consecutive salt-based repetitions of genes, old cell phones, circuit analysis, and inverse matrix calculator. The teacher used a problem-based learning approach combined with an online programming system to implement the STEM programming assignments to develop students’ STEM cross-domain knowledge, collaboration, and STEM programming skills, as shown in Figure 2.

As shown in Figure 2, the teacher used the problem-based learning approach and an online programming system to provide STEM curriculum materials and STEM programming assignments for the online activity. Since the online programming system was a self-learning system, students submitted their STEM programming assignments through the system and selected the type of compiler and code submission method. After submission, the system immediately compiled and responded to the students with the results. If there was an error in the submitted code, students would get a message on the page and would know where the code error was. They would know the teacher’s simple example hint for the STEM program assignments on the submission page. Through the online programming system, the teacher was able to know the students’ learning status such as the number of programming assignments submitted, successful or unsuccessful programming code submissions, and other operational events.

The teacher implemented weekly STEM programming assignments. Students followed a step-by-step problem-solving process in the problem-based learning approach: Stating the problem, understanding the problem, developing a solution plan, and executing the plan, reflecting, and debugging.

The first step was to state the problem: The teacher explained the objective of the STEM programming assignment and made sure that the students knew the key points of the assignment and the possible limits of the assignment. For example, the teacher explained the scientific conceptual programming assignment for the number of consecutive salt-based repetitions of genes. The teacher explained the main point of this assignment, which is “Please enter a DNA string consisting of four salts (A, T, C, and G) and an integer n. Please find all the repetitions of this DNA string with n consecutive salt groups.”

The second step was to understand the question: They needed to clarify the information in the question and understand and confirm the input and output requirements of the STEM programming assignment. For instance, the teacher explained the input and output requirements of the scientific conceptual programming assignment for the number of consecutive salt-based repetitions of genes. The input description was “Each input has a DNA string and a positive integer n.” The output description was “The number of repetitions of n consecutive salt groups according to the entered DNA string and the positive integer n.”

The third step was to develop a solution plan: When the student understood the input and output requirements of the STEM programming assignment, the teacher could guide the student to develop a step-by-step solution plan and execution time, listing the goals to be achieved in each phase and a check schedule. The teacher could provide code passages for students to fix missing parts and add breakpoints to help students reduce debugging time in the development process. The plan must remain flexible enough to allow for the possibility that the solution period may be extended or shortened for some unpredictable reason.

The fourth step was to execute the plan, reflect and debug: Students executed this solution plan, took a practical test, confirmed the correctness of the solution, and reflected on the correction of errors. If a student encountered a problem, the teacher gave tips on how to fix the code according to the solution plan. During this step, the teacher observed the student’s log of program debugging to understand the student’s learning, and provided timely guidance to give hints on how to solve the STEM programming assignment.

At the end of the experimental activity, the teacher conducted a posttest to understand the students’ learning effectiveness and administered a learning perception questionnaire. Finally, we collected the data from the tests, questionnaires, and the system logs of the online programming system for subsequent experimental analysis.

### Instruments

#### Test

The pretest and posttest were the same test. The test was designed by experienced STEM programming teachers (All et al., 2017; Chen et al., 2019). The pretest and posttest comprised 10 multiple-choice questions, each worth 10 points, giving a total of 100 points. The questions on the test contained basic programming knowledge, computer numerical computation, and STEM programming skills. The pretest measured students’ prior knowledge level of STEM programming. The posttest was to evaluate students’ learning effectiveness in the online STEM programming course. The pretest and posttest had greater than 50% difficulty, which means that they were appropriate for determining the level of students’ STEM programming skills.

We used the test results to classify the low scoring group (the bottom 33%) and the high scoring group (the top 33%). Based on the low and high score groups, we calculated the difficulty and discrimination of the pretest and posttest (Jonassen et al., 1993; Jonassen and Grabowski, 1993). The difficulty of the pretest was 52%, while the discrimination of the pretest was 30%. The difficulty of the posttest was 69%, while the discrimination of the posttest was 38%. The difficulty level of the pretest and posttest was greater than 50%. This means that the test was appropriate for determining the level of students’ STEM programming skills. The discrimination between the pretest and posttest was greater than 30%, which means that the test had good questions.

#### STEM learning perception questionnaire

We modified the learning perception questionnaire of previous studies (Wild and Schiefele, 1994; Griese et al., 2015) to create a STEM learning perception questionnaire. This questionnaire was used to understand students’ STEM learning perceptions using the problem-based learning method and the online programming system integrated into the online STEM programming course. The questionnaire’s dimensions were divided into organization, elaboration, metacognition, effort, attention, and reference. The organization dimension was used to explore students taking notes in the course. The elaboration dimension was used to explore the associations and integration of STEM themes in student learning in the course. The metacognition dimension was used to explore how students acquired STEM programming knowledge in the course. The effort dimension was used to explore students’ goals and expectations for the course. The attention dimension was used to explore the level of attention and concentration of students in the course The reference dimension was used to explore the use of reference resources in this course to assist students in learning STEM programming knowledge. The questionnaire comprised six questions in the organization dimension, eight questions in the elaboration dimension, six questions in the metacognition dimension, six questions in the effort dimension, six questions in the attention dimension, and four questions in the reference dimension, for a total of 36 questions.

The questionnaire was administered using a 5-point Likert-type scale. We assessed the reliability of the questionnaire using the Cronbach’s alpha coefficient method (Cohen, 1988). The Cronbach’s alpha coefficients of the organization, elaboration, metacognition, effort, attention, and reference dimensions were 0.91, 0.95, 0.89, 0.89, 0.96, and 0.95, and all six dimensions had acceptable reliability. Finally, the Cronbach’s alpha coefficient of the whole questionnaire was 0.94, which was higher than 0.7, meaning that the questionnaire had high reliability.

## Results

### Analysis of students’ prior knowledge and learning outcomes

The pretest measured differences in prior knowledge among students, while the posttest measured the students’ learning effectiveness. We compared the differences in students’ prior knowledge and learning outcomes.

The mean of the pretest was 51.00, and the standard deviation was 11.54. From the standard deviation in the pretest, we know that the low-scoring group of students had no experience of STEM programs. The mean of the posttest was 71.75 and the standard deviation was 12.27. This result indicated that the average score of students on the posttest was higher than the pretest.

We applied the paired sample *t* test to compare the pretest with the posttest. This result showed that the value was-11.16 and the two-tailed test was significant (*p* < 0.001), rejecting the null hypothesis. We found that Cohen’s *d* was 0.891, which is an effective value to achieve high efficiency (Cohen, 1988; Lenhard and Lenhard, 2016). This result shows that there was a significant difference between students’ prior knowledge and learning outcomes.

Students were divided into the high and low prior knowledge groups based on their pre-tests. The low prior knowledge group (10 students) was 50 points below the mean of the pretest, while the high prior knowledge group (10 students) was 50 points above the mean of the pretest. We used the paired-sample *t* test to analyze the learning effectiveness of the high and low prior knowledge groups, as shown in Table 1. There was a significant difference in the learning outcomes of students in the high prior knowledge group (*t* = −11.21, *p* < 0.001). Furthermore, there was a significant difference in the learning outcomes of students in the low prior knowledge group (*t* = −6.57, *p* < 0.001). This indicated that most students’ learning outcomes were higher than their prior knowledge.

**Table 1:** The result of the learning effectiveness of the high and low prior knowledge groups.

Group	Item	Prior knowledge	Learning performance	*t*	*p*
Low prior knowledge group (10 students)	MIN	30.00	45.00		
	MAX	50.00	80.00		
	Mean	43.00	65.00		
	S.D.	6.75	12.69	−6.57	0.000***
High prior knowledge group (10 students)	MIN	50.00	70.00		
	MAX	80.00	95.00		
	Mean	59.00	78.50		
	S.D.	9.66	7.47	−11.21	0.000***

***p < 0.001.

### Analysis of students’ STEM learning perceptions

In this study, we explored students’ STEM learning perceptions using problem-based learning methods and online programming systems integrated into the online STEM programming course. We used the one sample *t* test to analyze the results of students’ responses to the STEM learning perceptions, as shown in Table 2. These results found that the organization dimension, elaboration dimension, metacognition dimension, effort dimension attention dimension, and reference dimension were significant, as shown in Table 2, rejecting the null hypothesis. Table 2 shows that there are significant differences in students’ responses to each dimension of the STEM learning perception questionnaire. The results show that the STEM learning perceptions of the students in the course were different from the STEM learning perceptions of the students in the previous course. This also means that the use of a problem-based learning approach with the existing programming system is helpful for students taking the online STEM programming course.

**Table 2:** The result of students’ STEM learning perceptions.

Dimension	Mean	*SD*	*t*	*p*
Organization	3.01	0.77	17.35	0.000***
Elaboration	3.18	0.84	16.76	0.000***
Metacognition	3.29	0.81	18.01	0.000***
Effort	3.43	0.87	17.46	0.000***
Attention	3.15	1.10	12.70	0.000***
Reference	3.93	0.80	21.94	0.000***

***p < 0.001.

### Analysis of students’ answering STEM programming assignment behaviors

#### The distribution of solving STEM programming assignment behavioral processes

Students submitted their STEM programming assignments using the online programming system and the total number of submissions was 707 times, as shown in Table 3. We processed the results of the STEM programming assignments and divided them into the correct answers behavior (105 times) and the wrong answers behavior (602 times). The correct answers behavior included Science (31 times), Technology (21 times), Engineering (31 times), and Mathematics (22 times). It was found that the number of engineering programming assignments was the highest, while the number of Science programming assignments was the lowest. The wrong answers behavior included Science (43 times), Technology (234 times), Engineering (260 times), and Mathematics (65 times). It was found that the greatest number of Science programming assignments were submitted while the least number of Technology programming assignments were submitted.

**Table 3:** The results of STEM learning perception and answering STEM programming assignment behaviors.

Behaviors	Organization	Elaboration	Metacognition	Effort	Attention	Reference
Wrong answers	0.14	0.45*	0.28	0.15	0.27	−0.10
Correct answers	0.52*	0.59**	0.52*	0.32	0.47*	−0.33

**p < 0.01, *p < 0.05.

Table 4 presents the behavioral processes of students with different prior knowledge groups solving STEM programming assignments. First of all, the number of wrong answers for the group with high prior knowledge (349 times) was found to be higher than that of the group with low prior knowledge (253 times). On the other hand, we found that the number of correct answers (57 times) was higher for the low prior knowledge group than for the high prior knowledge group (48 times). In addition, we found that the number of responses from students tended to decline as the duration of the activity lengthened. After students became familiar with the problem-based learning approach and online programming system, most of them repeatedly watched the teacher’s problem-solving skills materials in the course. Students had a deep knowledge of problem-solving skills and designed different problem-solving plans to pass the code validation of STEM programming assignments for better learning performance.

**Table 4:** The distribution of solving STEM programming assignment behavioral processes.

Behaviors	Prior knowledge group	Science	Technology	Engineering	Mathematics	Total
Wrong answers (times)	Low	20	102	93	38	253
	High	23	132	167	27	349
	Total	43	234	260	65	602
Correct answers (times)	Low	13	12	20	12	57
	High	18	9	11	10	48
	Total	31	21	31	22	105

#### Analysis of relationships between solving STEM programming assignment behaviors and STEM learning perceptions

According to Landis and Koch (1977), we used the Spearman’s Rank Correlation Coefficient method to analyze whether there was a significant correlation between answering STEM programming assignment behaviors and students’ responses to each dimension of the STEM learning perception. In Table 3, we find that there is a significant correlation between the wrong answers behavior and the Elaboration dimension. In addition, we found that the Organization, Elaboration, Metacognition, and Attention dimensions were significantly correlated with the correct answers behavior.

## Discussion and conclusion

In this study, we used a problem-based learning approach with an online programming system to integrate an online STEM programming activity. After the experimental activity, we collected the experimental data and analyzed them. We explored the effects of using the problem-based learning approach with the online program system integrated into an online STEM programming curriculum on students’ learning outcomes, STEM learning perceptions, and learning behaviors.

### Prior knowledge and learning outcomes

After statistical analysis of the pre-test and post-test, we found that there were significant differences in students’ prior knowledge and learning outcomes. This means that most students used the problem-based learning method and an online programming system to help them learn STEM programs, which in turn led to a significant increase in their learning performance.

Next, we divided the group into a high and a low prior knowledge group. After statistical analysis of the high and low prior knowledge groups’ learning performance, it was found that there was a significant difference between the two groups’ learning outcomes. Regardless of whether it was the high prior knowledge group or the low prior knowledge group, students in both groups had good learning performance in the STEM programming activity. In the low prior knowledge group, students had a more significant increase in learning outcomes. We found that students focused on STEM programming skills and did not care about the theoretical knowledge of STEM programming, which led to the limitation of students’ learning effectiveness. Such results are similar to the problems encountered by students as mentioned by Achilleos et al. (2019), who indicated that students did not use basic STEM knowledge in their thinking patterns during the implementation of the program questions, resulting in limited learning.

Finally, this study used a problem-based learning approach with an online programming system to effectively enhance students’ STEM programming skills and further improve their learning outcomes. Our results are in line with Chonkaew et al. (2016) and Tang et al. (2020) who mentioned that the problem-based learning approach is helpful for students’ learning effectiveness.

### Students’ STEM learning perceptions

The STEM learning perception questionnaire includes the organization, elaboration, metacognition, effort, attention, and reference dimensions. The results revealed that there was a significant difference in students’ responses to each dimension of the STEM learning perception questionnaire. This means that using the problem-based learning approach with the online programming system is helpful for students taking STEM programming courses. In addition, we found that the mean of the organization dimension was slightly lower than others. We think that students do not like the idea of using notes to record classroom content. Instead, they enjoy working with their hands to identify problems and solve them to complete STEM programming assignments. In addition, it was found that the reference dimension was higher than other dimensions. We believe that when students encounter a problem while working on a STEM programming assignment, they will look at the textbook again and again to find a possible solution to the problem. When students do not understand the learning material in the online course, they will actively look for other online resources to learn possible problem-solving techniques. Nugent et al. (2015) mentioned that when students encounter problems in the classroom, they do not give up and are willing to continue learning to complete the STEM programming assignments, which is consistent with the similar results we found.

### Students’ answering STEM programming assignment behaviors

From the results of the STEM programming assignments submitted by the students of different prior knowledge groups, we found that there were differences in the students’ behaviors in answering the STEM programming assignments. It was found that the number of wrong answers of the high prior knowledge group was higher than that of the low prior knowledge group. On the other hand, the number of correct answers was higher for the low prior knowledge group than for the high prior knowledge group. We found that the students of the low prior knowledge group repeatedly confirmed the correctness of their STEM programming assignments. Furthermore, in the high prior knowledge group, students did not give up and continued to try possible solutions to complete their STEM programming assignments.

We then used the Spearman’s Rank Correlation Coefficient method to analyze students’ answering STEM programming assignment behaviors and their STEM learning perceptions. In this result, we see that there is a significant correlation between the wrong answers behavior and the Elaboration dimension of the STEM learning experience. This finding revealed that when students gave incorrect answers to the STEM programming assignments, they repeatedly looked at the learning materials in the online course. Students associated them with the problem solving skills taught by the teacher in the past, and tried different solutions until they completed the assignments. Our findings are similar to those of Lee et al. (2021). In other results, there was a significant correlation between the Organization, Elaboration, Metacognition, and Attention dimensions and the correct answers behavior. In terms of the Organization and Metacognition dimensions, we found that the students were in the habit of taking notes. This would help them to easily associate and integrate the STEM cross-domain knowledge learned in the online course and enhance their practical programming abilities. In terms of the Elaboration and Attention dimensions, students actively and intensely looked at the problem-solving skills in the learning materials repeatedly, and tried different problem-solving plans to complete the code validation of the STEM programming assignments. This result was consistent with the findings of Huang et al. (2020), who suggested that students might want to work hard to complete the STEM programming assignments to obtain good learning performance.

### Limitations and future study

Under the influence of COVID-19, remote learning was widely adopted in universities. In this study, a problem-based learning approach was combined with an existing online programming system for an online STEM programming activity. However, there are still shortcomings that need to be remedied in future research. First, this study only focused on the learning behaviors associated with an existing online programming system. In future studies, learning behaviors that affect college students’ online learning of STEM programming, such as many learning behaviors related to the features of the existing online programming system, can be discovered. In addition, the sample size should be expanded and the results should be examined with a broader and more representative sample in the future. Finally, this study lacked a comparison between an experimental group and a control group in order to present the differences in learning performance between the implementation of different teaching strategies and different teaching tools in the online STEM programming course. Future research may implement different teaching strategies and different experimental teaching tools to compare students’ learning performance in the online STEM programming course.

## Author contributions

All authors listed have made a substantial, direct, and intellectual contribution to the work and approved it for publication.

## Funding

This study was supported by the Ministry of Science and Technology, Taiwan, under two government grants (MOST 111-2410-H-019-006-MY3, MOST 111-2622-H-019-001, and MOST 109-2511-H-019-004-MY2).

## Conflict of interest

The authors declare that the research was conducted in the absence of any commercial or financial relationships that could be construed as a potential conflict of interest.

## Publisher’s note

All claims expressed in this article are solely those of the authors and do not necessarily represent those of their affiliated organizations, or those of the publisher, the editors and the reviewers. Any product that may be evaluated in this article, or claim that may be made by its manufacturer, is not guaranteed or endorsed by the publisher.
